# Malaria research challenges in low prevalence settings

**DOI:** 10.1186/1475-2875-11-353

**Published:** 2012-10-25

**Authors:** Gillian Stresman, Tamaki Kobayashi, Aniset Kamanga, Philip E Thuma, Sungano Mharakurwa, William J Moss, Clive Shiff

**Affiliations:** 1 Department of Immunology and Infection, London School of Hygiene & Tropical Medicine, London, W1CE 7HT, UK; 2 Department of Epidemiology, Johns Hopkins Bloomberg School of Public Health, Baltimore, MD, 21205, USA; 3 Faculty of Public Health and Policy, London School of Hygiene & Tropical Medicine, London, W1CE 7HT, UK; 4 Malaria Institute at Macha, Choma, Zambia; 5 Department of Molecular Microbiology and Immunology, Johns Hopkins Bloomberg School of Public Health, Baltimore, MD, 21205, USA

## Abstract

The prevalence of malaria has reduced significantly in some areas over the past decade. These reductions have made local elimination possible and the research agenda has shifted to this new priority. However, there are critical issues that arise when studying malaria in low transmission settings, particularly identifying asymptomatic infections, accurate detection of individuals with microparasitaemic infections, and achieving a sufficient sample size to have an adequately powered study. These challenges could adversely impact the study of malaria elimination if they remain unanswered.

## Background

After the failed eradication attempt of the 1950s, the malaria research objective was clear: reduce morbidity and mortality rates in areas where malaria persisted. These objectives made malaria relatively easy to study as areas with high morbidity and mortality had a high prevalence of infection as well as high parasite densities within infected individuals [1-4]. The focus on mortality reduction led to the development and widespread use of important tools to fight malaria, such as insecticide-treated bed nets, rapid diagnostic tests, and artemisinin, a drug effective against asexual parasitaemia and immature gametocytes, all of which have likely contributed to the reductions in malaria transmission in some areas [5-7]. The dramatic decline in malaria transmission has led to a shift from control to elimination as the primary goal [8]. This new malaria research agenda has led to new challenges: the populations of interest now include those that experience a low prevalence of infection and consist of asymptomatic and microparasitaemic individuals that may be beyond the limit of detection with current diagnostic tools. The ability to accurately study populations that are likely responsible for sustaining malaria transmission from one season to the next will be critical to achieving elimination, but are particularly difficult to assess with the diagnostic and epidemiological tools currently available [9,10]. Therefore, it is essential to discuss the current methodological barriers to the study of malaria in settings of low parasite prevalence and density, and the potential implications for confirming malaria elimination.

### Targeting asymptomatic infections

In areas where malaria is sustained at low levels or is highly seasonal, asymptomatic or minimally symptomatic reservoirs of infection are critical for maintaining malaria transmission as the parasite can persist in these individuals from one season to the next [11,12]. These reservoirs can comprise more than one third of a population [13] and have inherent challenges that make them difficult to identify, including the fact that these individuals do not seek treatment [14]. However, locating asymptomatically infected people and clearing this parasite reservoir will be critical to sustainable local and national malaria elimination [15,16]. In addition to finding ways to detect and prevent importation of malaria, as well as effective vector control measures, if control programmes ignore the local parasite reservoirs in human population’s malaria will likely resurge once control efforts are scaled back. A strategy that is simple and cost-effective in targeting gametocyte reservoirs is needed that can easily be integrated into local malaria control programmes.

Malaria infections tend to cluster in geographically defined areas with a higher malaria transmission, or hotspots of infection, during the low transmission season. These clusters that likely serve as the source of parasites that fuel subsequent transmission seasons [11]. The concept of hotspots could be used to target parasite reservoirs effectively and achieve maximum impact for control programmes. For example, research conducted in Zambia demonstrated that RDT-confirmed cases of malaria presenting at health facilities during the low transmission season could be used to target reservoirs of asymptomatic infection [17]. However, this approach may lead to sub-optimal coverage and may not be an efficient strategy in areas of low transmission intensity [18]. Other possible methods to target reservoirs could include using school surveys to identify possible hotspots of infection in the community or adults as a sentinel population [19]. If the defining characteristics of a hotspot of malaria infection could be clearly identified using either climate, entomological, or geographical data, these indicators could serve as a powerful tool to inform programmes on where to target control measures [20].

### Detection of low levels of parasitaemia

Even with the existence of a valid method to easily detect hotspots of malaria infection, including asymptomatic populations, a second limitation is our ability to diagnose infected individuals accurately. It is not uncommon for individuals to be infected with parasites that are sustained at microparasitaemic levels. The ability of current diagnostic tools to detect infections when parasite densities are low is poor and individuals negative for malaria by RDT or microscopy are still infectious to mosquitoes. [21] The commonly used tools for malaria diagnosis for research purposes include microscopy, rapid diagnostic tests, measuring the immune response to plasmodium (serology), and various methods of DNA detection including polymerase chain reaction (PCR), loop-mediated isothermal amplification (LAMP), or nucleic acid sequence-based amplification (NASBA) [22]. Although microscopy and RDT are the most commonly used methods of diagnosis, PCR can detect sub- patent infections and is frequently used for research purposes. [23,24]. However, the validity of PCR has not been adequately assessed for asymptomatic or microparasitaemic individuals: with a negative PCR result, what is the probability that a person is truly negative or is the negative due to insufficient sensitivity of the test? The analytical sensitivity of a nested PCR is often cited as being able to detect as few as two parasites per milliliter of whole blood [25]. Although the lower limit of detection is impressive, the consistency of the results in microparasitaemic individuals has never been properly assessed, especially in field settings where sample collection and storage may not be optimum. Microparasitaemic individuals produce gametocytes and likely contribute to the infectious reservoir [14]. However, individuals with infections below the sensitivity of PCR have received little attention and the probability that these individuals will produce and transmit gametocytes to mosquitoes, as well as their contribution to the infectious reservoir, has not been addressed.

### Power to detect the effectiveness of interventions

If it is possible to develop feasible strategies to target asymptomatic populations, and methods exist that can accurately identify those with low levels of parasitaemia, a third challenge provides a more fundamental problem: conducting valid epidemiological studies of malaria elimination in the field. The low transmission season is a critical bottleneck for targeting malaria reservoirs that sustain transmission from one season to the next, but parasite prevalence during this period can be extremely low. [11] Similarly, areas that have reduced transmission can experience extremely low parasite prevalence throughout the year. Both of these circumstances render these regions ideal for elimination campaigns. However, when any disease is uncommon, irrespective of the study design used, epidemiological studies become difficult because of challenges in reaching the desired sample size to achieve adequate power to minimize the probability of wrongly accepting that the null hypothesis is true, known as type II error. For example, a recent cluster-randomized trial carried out in Tanzania to determine the effect of mass drug administration on malaria transmission failed to detect a significant effect between the intervention and control arms because the incidence of malaria was low in the control arm [26].

Achieving the desired sample size and adequate study power is essential for conducting conclusive studies. For some surveys, the challenge of sample size could be overcome by increasing the number of people, increasing the duration of sample collection or survey a broader geographical area. These alternatives come with additional costs and possible challenges of changes in seasonality or variation in the micro epidemiology having an impact on the desired outcome [27,28]. Other options include alternative study designs. For example, the use of a pre/post design or the absence of a control population are not common in malaria epidemiology research because traditional epidemiological practice requires a design that allows the relative impact to be measured in reference to a comparison group. However, if malaria is eliminated in a population and the absence of infection confirmed by the absence of malaria antibodies circulating in the blood or other definitive test such as a PCR assay that is both sensitive and demonstrates consistent results, then the effectiveness of the approach would have been demonstrated. The ability to study malaria with sufficient power in elimination settings will be critical to provide insight to the best approaches, their effectiveness and to identify the significance of importations of parasites using molecular epidemiology.

### Implications for malaria elimination

The presence of asymptomatic infections, the inability to accurately diagnose microparasitaemic infections and gametocytemia, and the difficulty in achieving adequately powered studies in the context of low malaria prevalence all have serious implications for malaria elimination campaigns. How is it possible to understand malaria in low prevalence settings if there is little confidence that the people reported to be negative by PCR are uninfected? If sufficiently powered studies are difficult or impossible to implement, is it possible to eliminate the probability of a null hypothesis? The World Health Organization (WHO) defines malaria as eliminated in an area if there is zero incidence of locally acquired clinical cases in a defined geographical area during three consecutive years [29]. In settings with large numbers of asymptomatically infected people, how can this be assessed without testing populations for serological evidence of recent exposure? Until sufficient measures for elimination are developed for use in areas with persistent pockets of asymptomatic or minimally symptomatically infected individuals, or a successful mass drug administration campaign or vaccine is implemented, how is it possible to claim that these malaria reservoirs are truly eliminated? Also, how perfect does the optimum diagnostic method need to be before there is confidence in the results in an elimination context? In other words, what is the implication for continued transmission if even just one person is misclassified as negative who is actually carrying parasites? For malaria elimination to be sustainable and stand a real chance for success, it is imperative that these fundamental questions in studying malaria in low prevalence settings are addressed while it is still possible for malaria elimination campaigns to succeed.

## Competing interests

The authors declare that they have no conflicts of interest.

## Authors' contributions

GS conceived and prepared the manuscript, and TK, AK, PT, SM, WM, and CS contributed to developing the concepts presented in this paper and provided valuable insight during the revision and editing stages. All authors read and approved the final manuscript.
